# Validation and performance assessment of a commercial anti-peroxidasin antibody

**DOI:** 10.1007/s00418-025-02453-7

**Published:** 2026-01-21

**Authors:** Isabel Brandão, Roberto Silva, Bárbara Gomes, Jorge R. Almeida, João Paulo Oliveira, Inês S. Alencastre

**Affiliations:** 1 Instituto de Investigação e Inovação em Saúde, Porto, Portugal; 2 Universidade do Porto – Instituto de Ciências Biomédicas Abel Salazar, Porto, Portugal; 3 Unidade Local de Saúde de São João, Porto, Portugal; 4 Universidade do Porto – Faculdade de Medicina, Porto, Portugal; 5 RISE-Health, Porto, Portugal

**Keywords:** Anti-PXDN antibody, Immunofluorescence, Human kidney biopsies, Kidney fibroblasts

## Abstract

Peroxidasin (PXDN) is a multi-domain heme peroxidase that catalyzes sulfilimine cross-links in type IV collagen, a reaction essential for basement membrane stability. In addition to its structural role, PXDN has been implicated in oxidative stress regulation, fibrosis, and tumorigenesis. Antibody-based detection is critical for defining PXDN expression and localization, yet few commercial reagents have been rigorously validated, limiting reproducibility. Here, we assessed the specificity and performance of a commercially available anti-PXDN antibody (Abbexa, abx101906) in immunohistochemistry and immunocytochemistry applications. Validation was performed in formalin-fixed, paraffin-embedded human kidney tissue and in a primary kidney fibroblast line under *PXDN* silencing and overexpression conditions. The antibody yielded reproducible labeling patterns and demonstrated specificity across cellular contexts. These findings establish abx101906 as a reliable tool for immunofluorescence-based detection of PXDN. This validation provides a foundation for future studies of PXDN biology in kidney development, disease, and potential therapeutic targeting.

**Supplementary Information:**

The online version contains supplementary material available at 10.1007/s00418-025-02453-7.

## Introduction

Peroxidasin (PXDN) is a unique glycoprotein of 1479 amino acids that combines a heme peroxidase domain with extracellular matrix–associated motifs, including leucine-rich repeats, immunoglobulin-like domains, and a von Willebrand factor type C domain (Nelson et al. 1994; Cheng et al. 2008; Cheng and Shi 2022). Its primary transcript encodes a 26-amino acid signal peptide that directs the nascent chain into the endoplasmic reticulum, where the peptide is cleaved during maturation. PXDN subsequently assembles into disulfide-linked homotrimers (Paumann-Page et al. 2020; Soudi et al. 2015) and undergoes further post-translational processing, most notably proteolytic cleavage of its C-terminus by proprotein convertases after Arg1336 (Colon and Bhave 2016; Kovacs et al. 2021), a modification that facilitates extracellular matrix incorporation and enhances enzymatic activity. Unlike its extracellular form, intracellular PXDN remains uncleaved. PXDN catalyzes the formation of sulfilimine (S=N) bonds between methionine and hydroxylysine residues, a reaction essential for the stabilization of type IV collagen networks within basement membranes (Bhave et al. 2012; Fidler et al. 2014; Ero-Tolliver et al. 2015; Lazar et al. 2015).

In addition to its canonical role in extracellular matrix cross-linking, PXDN has emerged as a molecule of growing biomedical interest due to its involvement in oxidative stress regulation, tissue remodeling, fibrosis, and tumorigenesis (Cheng and Shi 2022). PXDN is widely expressed in human tissues, including the kidney, heart, and vasculature, and localizes to both secreted and membrane-associated compartments within cells (Peterfi et al. 2009).

Antibody-based techniques such as immunofluorescence (IF) are crucial for characterizing protein expression, post-translational modification, subcellular localization, and molecular interactions. Although commercial anti-PXDN antibodies are available, few have been rigorously validated across multiple applications. Many published studies have relied on custom-made or insufficiently characterized reagents, limiting reproducibility and translational insight. Furthermore, antibodies often vary substantially in specificity, affinity, and performance across assay types and species. In preliminary work, we evaluated several different commercial anti-PXDN antibodies in formalin-fixed, paraffin-embedded (FFPE) kidney tissue samples, identifying two that produced consistent and reproducible results.

In this study, we assessed the specificity and performance of a commercially available anti-PXDN antibody (Abbexa; Cambridge, UK; catalog # abx101906) in chromogenic (3,3′-diaminobenzidine [DAB]-based staining) and IF-based immunohistochemistry, across distinct sample preparation methods and cellular contexts. Validation followed key principles of antibody validation (Hewitt et al. 2014; Uhlen et al. 2016), employing human kidney tissue and a human kidney fibroblast primary cell line under PXDN silencing and overexpressing conditions, thereby enabling robust evaluation of signal specificity and background reactivity. The kidney tissue was selected as the primary biological sample in this study owing to the increasing recognition of PXDN in kidney physiology and pathology—a main focus of our research work—that remains yet underexplored.

By validating tools for the immunodetection of PXDN, this study provides a foundation for systematic investigation of its roles in development, disease, and potential therapeutic targeting.

## Materials and methods

### Antibody details

The rabbit polyclonal anti-PXDN antibody abx101906 is raised against a recombinant human PXDN peptide (Glu76–Gly260) expressed in *Escherichia coli* and purified by antigen-specific affinity chromatography, followed by protein A affinity chromatography. A homology search using the Basic Local Alignment Search Tool (BLAST^®^; https://blast.ncbi.nlm.nih.gov/Blast.cgi) confirmed that the peptide used as the immunogen is unique to PXDN. For independent orthogonal validation, a second commercially available polyclonal anti-PXDN antibody (Abbexa; Cambridge, UK; catalog # abx240259), raised in rabbits against human PXDN and purified by immunogen affinity chromatography, was also employed. Details of all primary and secondary antibodies used in this study are listed in Table 1.

**Table 1:** Details of the primary and secondary antibodies

Antibody	Immunogen	Host	Clonality	Manufacturer	Catalog number
Peroxidasin homolog (PXDN) antibody	Recombinant human PXDN protein (Glu76-Gly260) expressed in E. coli)	Rabbit	Polyclonal	Abbexa	abx101906
Peroxidasin homolog (PXDN) antibody	Human PXDN homolog	Rabbit	Polyclonal	Abbexa	abx240259
Anti-rabbit IgG (H+L), Alexa Fluor™ Plus 647	Gamma immunoglobulin heavy and light chains	Donkey	Polyclonal	Invitrogen	A-31573

### Kidney tissue immunohistochemistry

Frozen (OCT Mounting Media for cryotomy, VWR International, Pennsylvania, USA, catalog # 00411243) and formalin‑fixed paraffin‑embedded (FFPE) specimens of mature human kidney tissue with normal histomorphological features (*n* = 5), obtained from nephrectomy specimens performed for kidney cancer, were retrieved from the archives of the CHUSJ Anatomic Pathology Department. All kidney samples included at least one intact glomerulus, and tissue from nephrectomies was required to be collected at a minimum distance of 1 cm from tumor margins. From each tissue block, 3-μm-thick sections were prepared and mounted onto glass microscope slides according to standard local laboratory procedures.

For chromogenic and immunofluorescence analyses, FFPE sections were deparaffinized in fresh xylene (3 × 10 min), rehydrated through a graded ethanol series (100%, 96%, 70% and 50%; 10 min each), and immersed in double-distilled water. After deparaffinization and rehydration, slides designated for PXDN immunodetection were subjected to heat-induced epitope retrieval in sodium citrate buffer (pH 6.0) at 95° for 20 min, then cooled at room temperature for an additional 20 min. Frozen tissue sections were processed directly as follows from this point on. Slides were washed in phosphate-buffered saline (PBS; pH 7.4) and incubated for 60 min at room temperature in blocking/permeabilization buffer containing 10% normal horse serum and 0.1% Triton X-100 in PBS.

### Chromogenic detection

Following quenching of endogenous peroxidase activity, the slides were incubated with the Lab Vision™ UltraVision™ Large Volume Detection System: anti-Polyvalent, HRP (horseradish peroxidase) (Thermo Fisher Scientific Anatomical Pathology, Cheshire, UK) IHC (immunohistochemistry) staining kit, using diaminobenzidine (Dako, Carpinteria, CA, USA) as the peroxidase chromogen, and counterstained with hematoxylin (Leica Biosystems, Richmond, IL, USA).

### Immunofluorescence

Following blocking, the slides were incubated overnight at 4 °C with the primary antibody solutions diluted in blocking buffer. After washing with phosphate-buffered saline (PBS), the slides were incubated for 1 h at room temperature with the secondary antibody solutions (Table 1), also diluted in blocking buffer. Subsequently, the slides were washed in PBS and incubated with 4′,6-diamidino-2-phenylindole (DAPI) solution in PBS for nuclear counterstaining. After thorough PBS washes, the slides were mounted with ProLong™ Gold Diamond Antifade Mountant (Thermo Fisher Scientific, Waltham, MA, USA; RRID: P36965), allowed to polymerize overnight at room temperature, and stored in the dark at 4 °C until analysis. Control slides, in which the primary antibody was omitted, were processed in parallel (Supplementary Figure S1).

### Image acquisition

Both chromogenic and immunofluorescence slides were digitized into high-resolution images using a NanoZoomer S60 Digital Slide Scanner (Hamamatsu Photonics; Hamamatsu, Japan) with a 40× objective (numerical aperture of 0.95). The camera model is the ORCA-Flash4 with integrated scientific CMOS (sCMOS) sensor (Hamamatsu, Japan) for fluorescence imaging. Image acquisition was conducted with NZAcquire software (version U14297-01). Brightfield images were captured at 8-bit per RGB channel, and fluorescence images were recorded in 8-bit grayscale, both at 0.23 µm/pixel resolution. For fluorescence imaging, DAPI and Cy5 filters integrated in NanoZoomer were used. All images were saved in.ndpi file format. Fluorescence intensity was quantified using ImageJ/Fiji version 25, following rolling ball-background subtraction.

### Peptide-blocking assay

Tissue fluorescence intensity was compared between kidney slides stained with the primary antibody as supplied by the manufacturer, and slides in which the primary antibody was pre-incubated with recombinant human peroxidasin homolog protein (Abbexa; RRID: abx068474), at equimolar (1:1) or fivefold molar excess concentration (Fig. 2). As a negative control for the nonspecific binding, slides were incubated with the secondary antibodies in the absence of the primary antibody (data not shown). Nuclei were counterstained with DAPI for 10 min at room temperature. Fluorescence images were acquired using a Leica SP5 confocal microscope (Leica DMI6000-CS, Leica Microsystems, Wetzlar, Germany) with a 40× objective, with a numerical aperture of 1.25 and oil immersion correction. The Leica SP5 system operates through laser-scanning detection architecture equipped with photomultiplier tube detectors for fluorescence imaging and transmitted-light detectors for brightfield imaging. The instrument includes an integrated acousto-optical beam splitter. Imaging software was the Leica Application Suite Advanced Fluorescence software, version 2.3.2 (Leica Microsystems, Wetzlar, Germany), at a resolution of 1024 × 1024 pixels and 16-bit depth. Fluorescence excitation and detection were carried out using a 405-nm diode laser for the Alexa Fluor DAPI channel and a 633-nm helium–neon laser for the Alexa Fluor 647 channel, both operated at 10% laser power. Acquisition parameters were kept constant across all samples.

### Immunofluorescence cytochemistry in human kidney fibroblasts under PXDN transient gene silencing and overexpression conditions

Primary HKF cells (Innoprot; Derio, Spain; catalog #P10666) were cultured under standard conditions until reaching approximately 80% confluence. Cells were then transfected using Lipofectamine™ RNAiMAX (Invitrogen, Thermo Fisher Scientific, Waltham, MA, USA; catalog #13778075), according to the manufacturer's protocol, with either Silencer PXDN siRNA (Ambion, Thermo Fisher Scientific; catalog #4392420) for PXDN silencing, or mirVana™ miRNA inhibitor hsa-miR-203 (Ambion, Thermo Fisher Scientific; catalog #4464084) for PXDN overexpression. Silencer Select Negative Control #1 siRNA (Ambion, Thermo Fisher Scientific; catalog #4390843) and mirVana™ miRNA Inhibitor Negative Control (Ambion, Thermo Fisher Scientific; catalog #4464076) were used as corresponding negative controls.

Following a 5-day incubation, two replicate HKF culture plates were processed: one for quantification of PXDN gene expression by reverse transcription quantitative real-time PCR (RT-qPCR), and the other for PXDN protein detection by immunofluorescence cytochemistry. For RT-qPCR, total RNA was extracted using the TripleXtractor Direct RNA Kit (Grisp, Porto, Portugal; catalog #GK23.0100), and reverse transcribed into cDNA using the PrimeScript RT Reagent Kit (TaKaRa Biotechnology, Shiga, Japan; catalog #RR014A), and *PXDN* expression levels were quantified relative to the 18S ribosomal RNA housekeeping gene using the 2^−ΔΔCT^ method.

For immunofluorescence cytochemistry, cells were washed twice with PBS, fixed with 4% paraformaldehyde for 20 min, and incubated for 60 min at room temperature with blocking/permeabilization buffer (10% normal horse serum and 0.1% Triton X-100 in PBS). Cells were then incubated overnight at 4 °C with either the abx101906 or the abx068474 anti-PXDN antibodies. As control for putative paraformaldehyde-induced cross-linking, the same procedure was followed for abx101906 staining in HKF cells fixated with cold 100% methanol for 10 min on ice. Donkey Anti-Rabbit IgG (H+L) Alexa Fluor™ Plus 647 (Invitrogen, Thermo Fisher Scientific; Waltham, MA, USA; catalog # A32795) was used as secondary antibody. Nuclei were counterstained with DAPI for 10 min at room temperature.

Fluorescence and brightfield images were acquired using a Leica SP5 confocal inverted microscope (Leica DMI6000-CS, Leica Microsystems, Wetzlar, Germany, detailed above) with a Leica HC Plan Apochromat Lbl. Blue 20× objective with a numerical aperture of 0.70 and oil immersion correction. Fluorescence intensity (arbitrary units, AU) was quantified in five randomly selected regions per condition using ImageJ software.

### Data analyses

Statistical analyses were performed using GraphPad Prism^®^ (GraphPad Software, Boston, MA, USA). Where appropriate, Tukey’s post hoc test for multiple comparisons was applied following one-way analysis of variance (ANOVA).

## Results

### Architectural and subcellular localization of PXDN expression

**Fig. 1:**
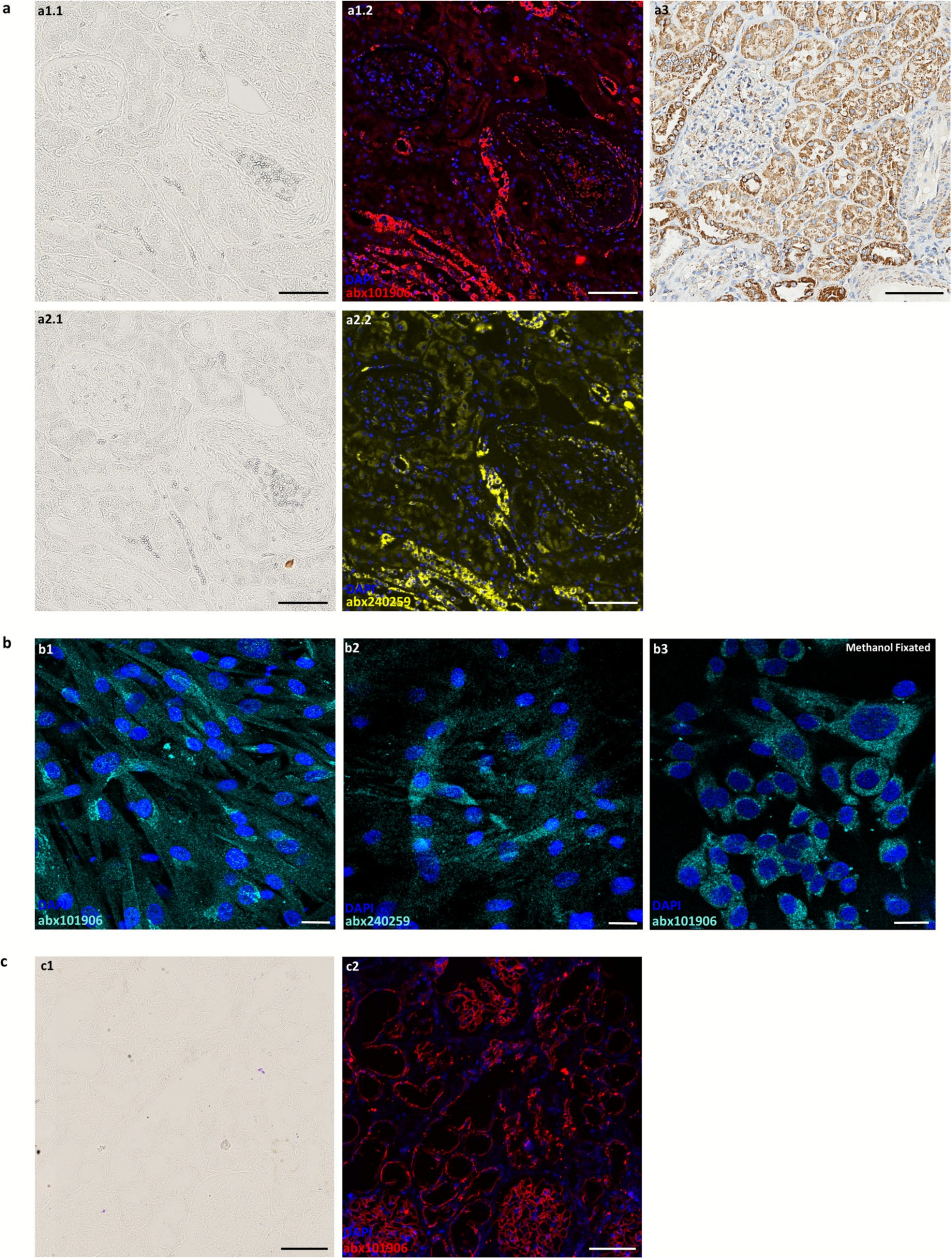
Representative immunohistochemical micrographs of FFPE and frozen human kidney tissue and human kidney fibroblast (HKF) cells showing peroxidasin (PXDN) expression using two distinct antibody staining methods. Brightfield and corresponding fluorescence images of 4′,6-diamidino-2-phenylindole (DAPI) and PXDN labeling using abx101906 and abx240259 in the FFPE kidney tissue [**a1** and **a2**, respectively]. Brightfield images of FFPE kidney tissue stained with abx101906 followed by quenching of endogenous peroxidase activity and incubated with the Lab Vision™ UltraVision™ Large Volume Detection System: anti-Polyvalent, HRP (horseradish peroxidase) (Thermo Fisher Scientific Anatomical Pathology, Cheshire, UK) IHC staining kit and counterstained with hematoxylin (Leica Biosystems, Richmond, IL, USA) [**a3**]. Fluorescence images of DAPI and PXDN labeling using abx101906 and abx240259 in paraformaldehyde-fixated HKF [**b1** and **b2**, respectively] and methanol-fixated HKF stained with abx101906 [**b3**]. Unstained brightfield and corresponding fluorescence images of DAPI and PXDN labeling using abx101906 in frozen human kidney tissue are displayed in [**c1**] and [**c2**], respectively. Details of the histology and staining methods are provided in Materials and Methods. The secondary antibodies used for the immunofluorescence staining were Donkey Anti-Rabbit IgG (H+L) Highly Cross-Adsorbed Secondary Antibody, Alexa Fluor™ Plus 647 (Invitrogen, Thermo Fisher Scientific; Waltham, MA, USA; catalog # A32795). All images from human kidney tissue were acquired in a NanoZoomer S60 Digital Slide Scanner (Hamamatsu Photonics; Hamamatsu, Japan). Scale bars: [**a**] and [**c**] 100 μm; [**b**] 20 μm. Color codes depicted in images—bottom left

IF and chromogenic staining with the anti-PXDN antibody abx101906 revealed a strong, punctate cytoplasmic pattern in FFPE human kidney tissue and in paraformaldehyde and methanol-fixed HKF cells (Fig. 1a1.2, a1.3, b1, b3). In FFPE kidney tissue, signal intensity was consistently higher in the tubular compartment compared with glomeruli and blood vessels. In frozen human kidney tissue, abx101906 additionally produced linear staining along glomerular and tubular basement membranes, in addition to the pattern observed in FFPE tissue (Fig. 1c2).

No signal was detected when the primary antibody was omitted, excluding nonspecific binding of the secondary antibody (Supplementary Figure S1).

### Orthogonal validation

Comparison of the abx101906 with the independently generated abx240259 anti-PXDN antibodies demonstrated similar fluorescence localization patterns in both human kidney biopsy samples (Fig. 1a1.2, a2.2, above) and HKF cells (Fig. 1b1, b2, above), supporting recognition of the same target protein in situ.

### Validation by peptide-blocking assay

**Fig. 2:**
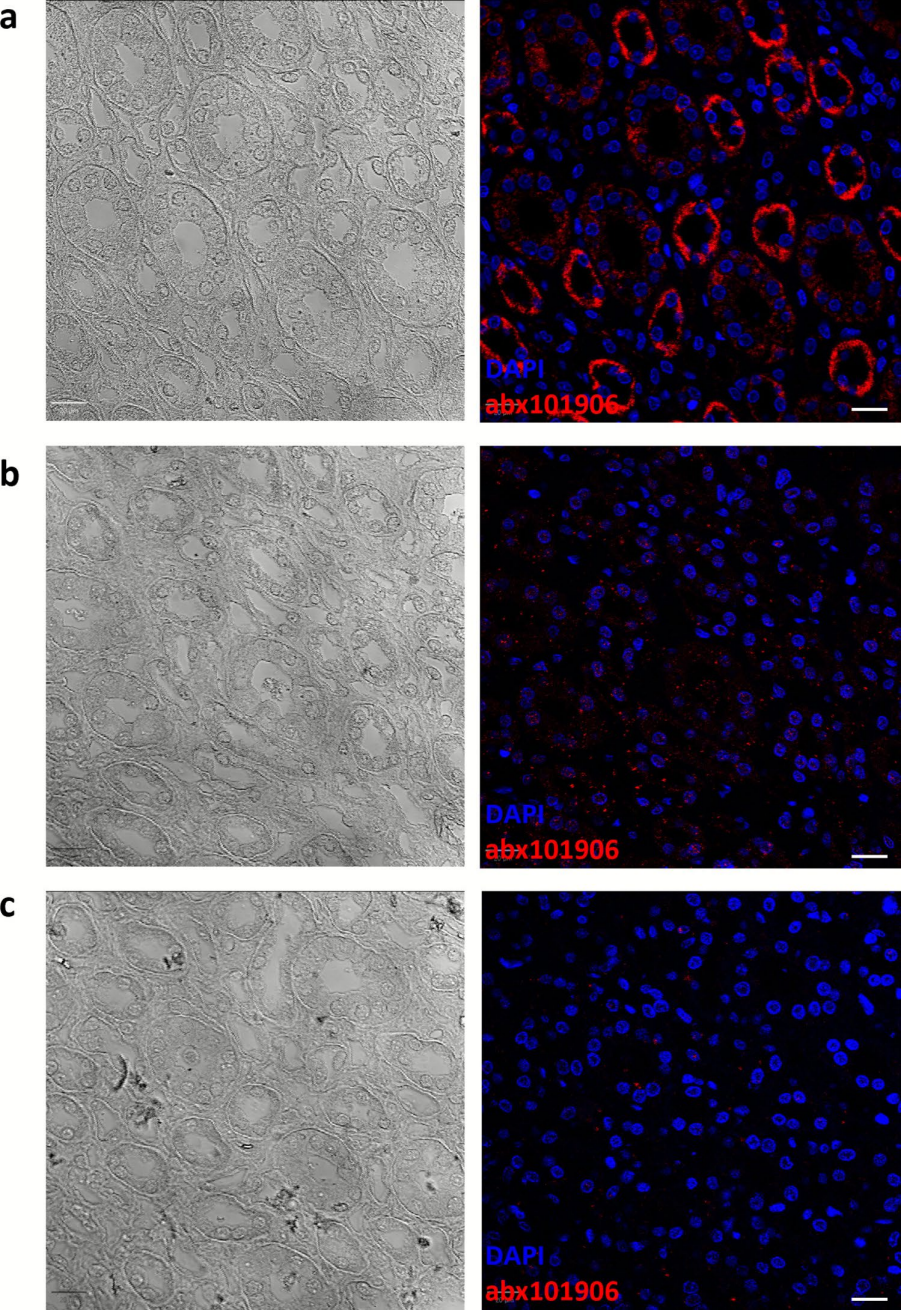
Validation of anti-peroxidasin (PXDN) antibody specificity: Competition assay. Adult human kidney 3-µm-thick tissue sections were stained with rabbit polyclonal anti-PXDN antibody (Abbexa; Cambridge, UK; catalog # abx101906), in the absence [**a**] and presence of recombinant Human Peroxidasin Homolog Protein (Abbexa; catalog # abx068474), in either 1:1 equimolar [**b**] or 5× molar excess [**c**] amounts—in the presence of the purified antigen, PXDN tissue staining was markedly reduced [**b**] or lost [**c**]. Donkey Anti-Rabbit IgG (H+L) Alexa Fluor™ Plus 647 (Invitrogen, Thermo Fisher Scientific; Waltham, MA, USA; catalog # A32795) was used as secondary antibody. Nuclei were stained with 4′,6-diamidino-2-phenylindole (DAPI) for 10 min at room temperature. Fluorescence and brightfield images were acquired in the NanoZoomer S60 Digital Slide Scanner (Hamamatsu Photonics; Hamamatsu, Japan). Scale bars: 20 µm. Color codes: DAPI nuclei staining, blue; PXDN staining, red

Pre-incubation of abx101906 with recombinant human PXDN markedly reduced fluorescence intensity in tissue samples. The signal was completely abolished when a fivefold molar excess of purified antigen was used for adsorption (Fig. 2). As expected, omission of the primary antibody yielded no detectable signal, confirming the absence of nonspecific secondary binding (data not shown).

### Validation by genetic methods

**Fig. 3:**
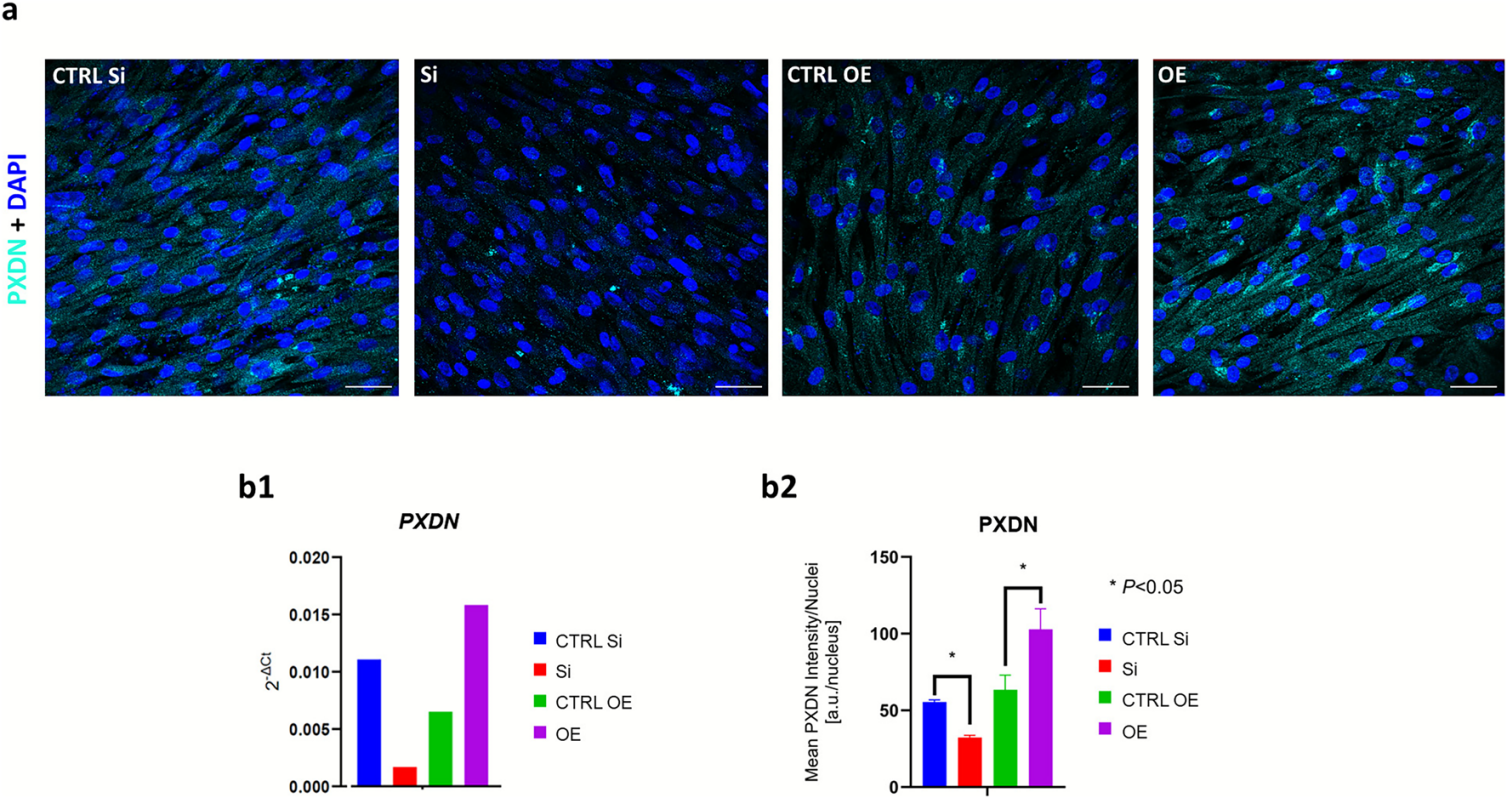
Validation of anti-peroxidasin (PXDN) antibody specificity: Immunofluorescence histochemistry in human kidney fibroblasts (HKF) under PXDN transient gene silencing and overexpression conditions. Primary HKF (Innoprot; Derio, Spain; catalog #P10666) were treated with either Silencer PXDN siRNA (Ambion, Thermo Fisher Scientific; Waltham, MA, USA; catalog #4392420) for PXDN silencing, or mirVana™ miRNA inhibitor hsa-miR-203 (Ambion, Thermo Fisher Scientific; Waltham, MA, USA; catalog #4464084), using Lipofectamine RNAiMAX (Invitrogen, Thermo Fisher Scientific; Waltham, MA, USA; catalog #13778075), according to the Lipofectamine™ RNAiMAX Transfection Reagent Thermo Fisher Protocol. Silencer Selected Negative Control #1 siRNA (Ambion, Thermo Fisher Scientific; Waltham, MA, USA; catalog #4390843) and mirVana™ miRNA inhibitor Negative Control (Ambion, Thermo Fisher Scientific; Waltham, MA, USA; catalog #4464076) were respectively used as negative controls for silencing and overexpression. Following incubation for 5 days of two replicate HKF culture plates, one was used for analysis of PXDN gene expression by reverse transcription quantitative real-time polymerase chain reaction (RT-qPCR), and the other for assessing PXDN protein expression by immunofluorescence cytochemistry. For the RT-qPCR assay, total RNA was extracted with the TripleXtractor direct RNA kit (Grisp; Porto, Portugal; catalog #GK23.0100), reverse-transcribed into cDNA using the PrimeScript RT reagent kit (TaKaRa Biotechnology; Shiga, Japan; catalog #RR014A), and the PXDN expression was quantified as its fold increase relative to the 18S ribosomal RNA housekeeping gene, using the 2^−ΔΔCT^ method. For immunofluorescence cytochemistry, cells were washed twice with PBS, fixed for 20 min with 4% paraformaldehyde, incubated with blocking/permeabilization buffer (10% normal horse serum and 0.1% Triton X-100 in PBS) for 60 min at room temperature, and incubated overnight at 4 °C in the same buffer, with rabbit polyclonal anti-PXDN antibody (Abbexa; Cambridge, UK; catalog # abx101906). Donkey anti-rabbit IgG (H+L) Alexa Fluor™ Plus 647 (Invitrogen, Thermo Fisher Scientific; Waltham, MA, USA; catalog # A32795) was used as secondary antibody. Nuclei were stained with 4′,6-diamidino-2-phenylindole (DAPI) for 10 min, at room temperature. Fluorescence images were acquired in a Leica SP5 Confocal microscope (Leica Microsystems) using the 40× oil objective (exemplary micrographs in [**a**]).  [**b1**]. Fluorescence intensity (arbitrary units (UA)) was retrieved in five randomly selected regions per condition, using ImageJ software [**b2**]. Scale bars: 50 µm. Color codes: DAPI nuclei staining, blue; PXDN staining, green. Legend: CTRL, Control; Si, *PXDN* silencing; OE, *PXDN* overexpression

In HKF cells, immunofluorescence cytochemistry under *PXDN* silencing or overexpression conditions—validated by RT-qPCR (Fig. 3 [**b1**])—showed a significant reduction of the PXDN fluorescence signal after silencing, and a marked increase following overexpression (Fig. 3 [**a**], [**b2**]).

## Discussion

First identified in the *Drosophila* nearly three decades ago (Nelson et al. 1994), PXDN has gained increasing attention for its roles in extracellular matrix stabilization, oxidative stress regulation, and disease pathogenesis (Cheng and Shi 2022). Despite this interest, many commercially available anti-PXDN antibodies lack the sensitivity and specificity required for reliable immunodetection, leading several groups to generate and validate in-house anti-PXDN antibodies (Cheng et al. 2008; Peterfi et al. 2009).

In this study, we evaluated the performance and specificity of the commercial anti-PXDN antibody abx101906 across multiple experimental platforms, following recommended validation principles (Hewitt et al. 2014; Uhlen et al. 2016). Our findings demonstrate that this antibody consistently detects PXDN, producing strong and specific cytoplasmic staining in human kidney tissue and cultured HKF cells. In human kidney tissue, staining was particularly enriched in renal tubular regions, suggesting compartment-specific expression. In HKF cells, the cytoplasmic signal persisted in both paraformaldehyde and methanol-fixed cells, indicating that it was not a fixation-related signal but reflects reliable intracellular localization.

Our data contrast slightly with the more discrete punctate extracellular matrix–associated labeling reported by Peterfi et al. (2009). This divergence is likely attributable to several methodological and biological factors. Peterfi and colleagues investigated PXDN in myofibroblasts using immunostaining optimized for detecting the secreted, matrix-incorporated form of the protein, whereas our study examined non-myofibroblastic primary kidney fibroblasts that express high levels of the intracellular, uncleaved precursor. In addition, differences in fixation protocols, antibody epitopes, and cellular activation states can substantially affect subcellular signal localization. Our findings therefore complement those of Peterfi et al. by highlighting that PXDN localization may vary according to the cell type, processing state, and methodological conditions, reflecting the dual intracellular and extracellular nature of the protein.

Antibody specificity was confirmed through peptide pre-adsorption and *PXDN* gene silencing, both of which abolished the signal. In addition, co-localization with an independently generated anti-PXDN antibody supported the selectivity of abx101906. A notable observation was the detection of PXDN along glomerular and tubular basement membranes in frozen sections, contrasting with the absence of basement membrane staining in FFPE sections, where only a cytoplasmic labeling was observed. Given that PXDN is an extracellular peroxidase that localizes to basement membranes to catalyze type IV collagen cross-linking, this discrepancy is likely attributable to sample processing. Formalin-induced cross-linking and subsequent dehydration/clearing steps compact the dense extracellular matrix, resulting in epitope masking and limited antibody penetration (Shi et al. 1991; Sompuram et al. 2004), whereas frozen sections preserve near-native antigen conformation and matrix accessibility. Consistent with this interpretation, heat-induced antigen retrieval with citrate (pH 6) or Tris–EDTA (pH 9) for 20 min at 97 °C failed to restore basement-membrane staining, suggesting that more aggressive or enzymatic retrieval approaches (e.g., proteinase K, pepsin, or titrated collagenase digestion) may be required to unmask PXDN epitopes in FFPE tissues.

Although the validation data collectively support the specificity of abx101906, potential cross-reactivity cannot be entirely excluded. As with all polyclonal antibodies, minor off-target binding may occur due to recognition of epitopes with partial sequence homology to unrelated proteins. However, several lines of evidence argue against significant cross-reactivity in this study. First, peptide blocking completely abolished the immunostaining signal, demonstrating that labeling was dependent on epitope recognition. Second, PXDN silencing markedly reduced signal intensity, whereas overexpression enhanced it, confirming that staining was linked to PXDN expression levels. Finally, the comparable localization patterns obtained using an independent anti-PXDN antibody (abx240259) further support antibody selectivity. While the possibility of low-level cross-reactivity cannot be fully ruled out, these combined data indicate that abx101906 displays high target selectivity and is suitable for reliable PXDN detection. Taken together, our results establish Abbexa abx101906 as a reliable tool for PXDN detection in tissue and cell immunofluorescence applications. This comprehensive validation provides a foundation for future studies of PXDN biology, including its roles in renal physiology and pathology, extracellular matrix remodeling, and oxidative stress-related diseases.

### Limitations

Despite the fact that polyclonal antibodies can exhibit lot-to-lot variability, this affinity-purified antibody targeting a unique amino acid sequence of PXDN showed robust performance; however, recombinant antibody formats could further enhance reproducibility in future studies.

## Supplementary Information

Below is the link to the electronic supplementary material.

Supplementary Figure S1 Control for nonspecific labeling. Brightfield [a1] and fluorescence images of formalin-fixed, paraffin-embedded human kidney tissue processed with the secondary antibody alone (Alexa Fluor 647) [a2]; as well as composite image of DAPI and Alexa Fluor 647 [a3]. No specific fluorescence signal was detected, confirming the absence of nonspecific secondary antibody binding. Scale bar: 100 μm. Color codes—DAPI nuclei staining, blue; Alexa fluor 647 staining, red (TIF 1882 KB)

## Data Availability

All data are included in the manuscript and supporting information.
