# Supplementary figures and images for: Validation and performance assessment of a commercial anti-peroxidasin antibody
Source: Histochem Cell Biol. 2026 Jan 21;164(1):5. doi: 10.1007/s00418-025-02453-7 (PMC12823669; doi:10.1007/s00418-025-02453-7)

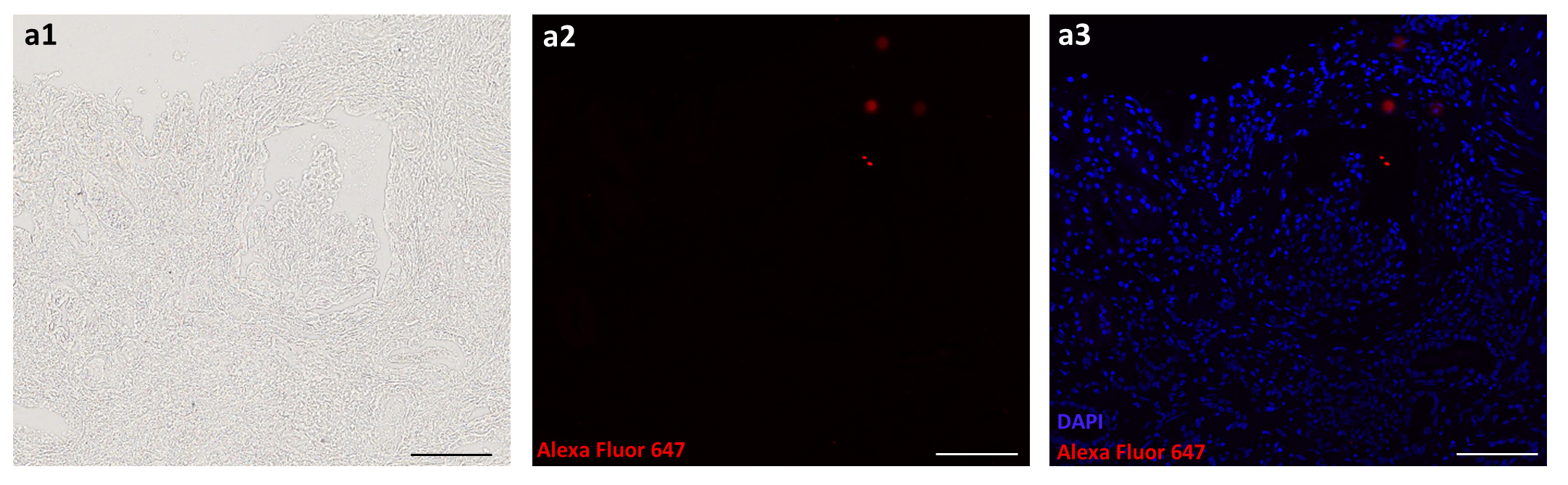

Supplement: Supplementary file 1 — Control for nonspecific labeling. Brightfield [a1] and fluorescence images of formalin-fixed, paraffin-embedded human kidney tissue processed with the secondary antibody alone (Alexa Fluor 647) [a2]; as well as composite image of DAPI and Alexa Fluor 647 [a3]. No specific fluorescence signal was detected, confirming the absence of nonspecific secondary antibody binding. Scale bar: 100 μm. Color codes—DAPI nuclei staining, blue; Alexa fluor 647 staining, red (TIF 1882 KB) [file 418_2025_2453_MOESM1_ESM.tif]
